# Prognostic Factors for Mortality in Patients With Autoimmune Diseases Complicated by Pneumocystis Pneumonia

**DOI:** 10.7759/cureus.67749

**Published:** 2024-08-25

**Authors:** Kunika Shimizu, Nobuyuki Yajima, Ryo Yanai, Kuninobu Wakabayashi, Yusuke Miwa, Takaaki Matsuyama

**Affiliations:** 1 Division of Rheumatology, Department of Medicine, Showa University School of Medicine, Tokyo, JPN; 2 Department of Legal Medicine, Showa University School of Medicine, Tokyo, JPN

**Keywords:** immunocompromised patient, rheumatology & autoimmune diseases, prognostic risk factors, pneumocystis jiroveci pneumonia, autoimmune rheumatic diseases

## Abstract

Objectives

Pneumocystis pneumonia (PCP) has a poor prognosis in patients with autoimmune diseases. Additionally, the mortality of PCP in autoimmune diseases is higher than that of human immunodeficiency virus-PCP. Therefore, this study aimed to assess the risk factors associated with mortality in patients with autoimmune diseases complicated with PCP.

Methods

We conducted a retrospective observational study involving 38 patients treated for PCP at Showa University School of Medicine from January 2010 to August 2014. Diagnoses were established based on clinical symptoms, imaging, and laboratory tests, including hypoxemia (partial pressure of oxygen (PaO2) < 70 mmHg), chest computed tomography findings, elevated (1,3)-β-D-glucan, and positive *Pneumocystis jirovecii*polymerase chain reaction tests from sputum samples. Data regarding patient demographics, underlying diseases, therapeutic interventions, and laboratory findings were collected. Statistical comparisons between survivors and non-survivors were performed using Fisher's exact probability test and Mann-Whitney U test for independence test with Stata/SE version 17.0 (StataCorp LLC, College Station, TX).

Results

The median age of the study group was 72.4 years old, with the majority having rheumatoid arthritis. Mortality occurred in 18% (seven out of 38) of cases. Factors associated with increased mortality include males, higher serum creatinine and C-reactive protein levels, lower albumin and immunoglobulin A levels, and lower PaO2/fraction of inspired oxygen (FiO2) ratio. No significant difference was observed in the rate of intratracheal intubation, ICU management, and hospitalization period. Prophylactic administration of trimethoprim-sulfamethoxazole (TMP-SMX) was not performed in any of the cases.

Conclusions

This study examined the risk of mortality for PCP in patients with autoimmune diseases. The male gender, low IgA and albumin levels, low PaO2/FiO2 ratio, as well as high creatinine and CRP levels, were identified as significant factors for poor prognosis. These factors can help identify patients at high risk for PCP and guide the consideration of prophylactic TMP-SMX administration. They may also provide insights into when to discontinue prophylactic treatment. Future studies should investigate the administration of prophylactic TMP-SMX. Additionally, the risk of mortality for PCP under the administration of new biological agents, such as anifrolumab, belimumab, and rituximab, or immunosuppressants, such as mycophenolate mofetil and voclosporin, should be evaluated. These findings can contribute to improving PCP prevention and treatment strategies in patients with autoimmune diseases, ultimately leading to better patient outcomes.

## Introduction

Recently, treatment for autoimmune diseases, including rheumatoid arthritis (RA) and systemic lupus erythematosus (SLE), has advanced with an improved prognosis for these diseases using glucocorticoids and immunosuppressants. However, complications associated with infectious diseases have become problematic. Half of the patients with RA and a quarter of those with SLE who died had infections, many of which were classified as opportunistic infections [1]. Pneumocystis pneumonia (PCP) is recognized because its mortality rate in autoimmune diseases is higher than that of human immunodeficiency virus (HIV)-PCP [2]. For example, the mortality rate of PCP associated with autoimmune diseases is approximately 6-62.5%, whereas the mortality rate of HIV-PCP is 6.7% [3,4].

The risk factors for PCP mortality included low levels of serum albumin and cholinesterase at the time of PCP diagnosis, decreased pulmonary diffusing capacity (widened A-aDO2), a significantly high rate of intratracheal intubation, and management in the intensive care unit (ICU) [5]. Additionally, other risk factors, such as lower lymphocyte counts, elevated (1,3)-β-D-glucan, hypoxemia, bilateral lung involvement in chest X-ray [6], and serum immunoglobulin (Ig) G level [7], have been identified, but no consensus is reached yet.

In this study, we evaluated these factors, previously reported poor prognostic factors, and assessed their value in cases of death from PCP. Hence, patients who should receive prophylactic administration of trimethoprim-sulfamethoxazole (TMP-SMX) are identified.

## Materials and methods

Study design and setting

We conducted a retrospective observational study based on the medical records of 38 patients with PCP complicated with autoimmune diseases who were admitted to the Division of Rheumatology, Department of Medicine, Showa University School of Medicine, between January 1, 2010, and August 31, 2014.

Patient selection

The following criteria were used to diagnose PCP: (1) dyspnea; (2) partial pressure of oxygen (PaO2) < 70 mmHg or (in room air) because of hypoxia, arterial blood gas, or medical necessity for oxygen therapy; (3) chest computed tomography presenting a mosaic pattern with normal secondary lobules adjacent to abnormal lobules [8]; (4) increased (1,3)-β-D-glucan [9]; and (5) a positive result for *Pneumocystis jirovecii* using sputum polymerase chain reaction (PCR) [10]. PCP was diagnosed when criteria (1), (2), and (3) were satisfied and either (4) or (5) were observed. Detecting bacteria using microscopy and staining was difficult because non-HIV-PCP involves fewer bacteria than HIV-PCP. Therefore, microscopic diagnosis was excluded.

Data collection

Additionally, information on background factors, including age, sex, baseline diseases, medications at the time of hospital admission, such as the dose of glucocorticoids and immunosuppressants, presence of complications, such as respiratory diseases, diabetes, and chronic kidney disease, smoking history, and TMP-SMX prophylaxis, were studied. For laboratory data, complete blood count (including lymphocyte count), serum lactate dehydrogenase (LDH), albumin, creatinine, C-reactive protein (CRP), KL-6, surfactant protein D (SP-D) [11], IgG [7], IgA, IgM, (1,3)-β-D-glucan, arterial blood gas, and sputum PCP deoxyribonucleic acid (tested via PCR) at the time of hospital admission were measured. We also assessed the treatment after hospitalization, including TMP-SMX used, change from TMP-SMX to pentamidine, pulsed glucocorticoid therapy, intratracheal intubation, ICU management, and hospitalization period.

Statistical analysis

The analysis was performed by comparing survivor and non-survivor groups. Fisher's exact probability test and Mann-Whitney U test were used, with a significance level of 5%. All statistical analyses were performed using Stata/SE software (version 17.0; StataCorp LLC, College Station, TX).

Ethics approval and informed consent

The Bio-Ethical Committee of the Showa University School of Medicine (No. 1644) approved this study. Written informed consent was obtained from the survivors and the families of the non-survivors. If the family did not provide informed consent, institutional review board approval was necessary to perform a retrospective study in accordance with the hospital policy. Based on the Bio-Ethical Committee of Showa University rules, any retrospective study that uses only the medical records of patients is subject to review by the Ethics Committee without the need for written consent from the patients. As this study included past cases of death, no written consent was obtained from the patients or their families.

## Results

Baseline characteristics of participants

Table 1 shows the baseline characteristics. The median age of the participants was 72.4 years old. Among the 38 patients included in this study, 28 (73.7%) had RA. Other autoimmune diseases included were SLE, systemic sclerosis, microscopic polyangiitis, Sjögren's syndrome, relapsing polychondritis pseudo gout, and autoimmune hepatitis. Furthermore, 15 (39.5%) patients had a history of smoking. Twenty-one (55.3%) and 11 (28.9%) patients had pulmonary involvement and diabetes mellitus, respectively. Death occurred in seven of 38 patients.

**Table 1:** Baseline characteristics of the participants. IQR: interquartile range; RA: rheumatoid arthritis; SLE: systemic lupus erythematosus; SSc: systemic sclerosis; SjS: Sjögren's syndrome; MPA: microscopic polyangiitis; RP: relapsing polychondritis; TMP-SMX: trimethoprim-sulfamethoxazole; MTX: methotrexate; TAC: tacrolimus; eGFR: estimated glomerular filtration rate; KL-6: Krebs von den Lungen-6; SP-D: surfactant protein D; PCR: polymerase chain reaction; ST: trimethoprim-sulfamethoxazole. ^a^ Fisher's exact probability test. ^b^ Mann-Whitney U test. * U statistic.

	Survivors (n = 31)	Non-survivors (n = 7)	p-value	Relevant test static
Male, n (%)	5 (16.1)	4 (57.1)	0.04^a^	-
Age, median (IQR)	71.9 (64.2, 79.6)	79.8 (71.5, 89.3)	0.08^b^	62*
Smoking history, n (%)	12 (38.7)	3 (42.9)	1.00^a^	-
Diabetes mellitus, n (%)	9 (29.0)	2 (28.6)	1.00^a^	-
Pulmonary disease, n (%)	15 (48.4)	6 (85.7)	0.10^a^	-
Baseline autoimmune disease			-	-
RA, n (%)	23 (74.2)	5 (71.4)	1.00^a^	-
SLE, n (%)	2 (6.5)	0 (0)	1.00^a^	-
SSc, n (%)	2 (6.5)	0 (0)	1.00^a^	-
SjS, n (%)	1 (3.2)	0 (0)	1.00^a^	-
MPA, n (%)	1 (3.2)	1 (14.3)	0.34^a^	-
RP, n (%)	1 (3.2)	0 (0)	1.00^a^	-
Pseudogout, n (%)	0 (0)	1 (14.3)	0.18^a^	-
Autoimmune hepatitis, n (%)	1 (3.2)	0 (0)	1.00^a^	-

Baseline characteristics of treatment and laboratory data at the onset of PCP

Table 2 presents the treatment and laboratory data at the onset of PCP. Prophylactic administration of TMP-SMX was not performed in any of the cases. The median prednisolone (PSL) dose at the PCP onset was 5 mg/day, and 30 (81.6%) patients were on immunosuppressants.

**Table 2:** Baseline characteristics of treatment and laboratory data at the onset of PCP. IQR: interquartile range; SSc: systemic sclerosis, SjS: Sjögren's syndrome; MPA: microscopic polyangiitis; RP: relapsing polychondritis; PCP: Pneumocystis pneumonia; TMP-SMX: trimethoprim-sulfamethoxazole; MTX: methotrexate; TAC: tacrolimus; eGFR: estimated glomerular filtration rate; KL-6: Krebs von den Lungen-6; SP-D: surfactant protein D; PCR: polymerase chain reaction; ST: trimethoprim-sulfamethoxazole; LDH: lactate dehydrogenase; CRP: C-reactive protein; PaO2/FiO2: partial pressure of oxygen/fraction of inspired oxygen; PCR: polymerase chain reaction; PSL: prednisolone. ^a^ Fisher's exact probability test. ^b^ Mann-Whitney U test. * U statistic.

	Survivors (n = 31)	Non-survivors (n = 7)	p-value	Relevant test static
Treatment at the onset of PCP				
PSL dose (mg/day), median (IQR)	5 (0, 10)	5 (5, 8)	0.89^b^	105*
Immunosuppressant				
MTX, n (%)	22 (71.0)	3 (42.9)	0.20^a^	-
TAC, n (%)	4 (12.9)	0 (0)	1.00^a^	-
Salazosulfapyridine, n (%)	0 (0)	0 (0)	1.00^a^	-
Biologics, n (%)	10 (32.3)	0 (0)	0.16^a^	-
Others, n (%)	2 (6.5)	1 (14.3)	0.47^a^	-
TMP-SMX prophylaxis, n (%)	0 (0)	0 (0)	1.00^a^	-
Laboratory data at the onset of PCP				
WBC count (/μL), median (IQR)	9100 (6800, 11800)	9800 (7100, 11800)	0.93^b^	106*
Lymphocyte count (/μL), median (IQR)	814.8 (529.2, 1150)	951 (649, 1377)	0.42^b^	130*
Hemoglobin (g/dL), median (IQR)	11.2 (10.4, 12.6)	11.5 (9.6, 12.3)	0.65^b^	120.5*
Serum creatinine (mg/dL), median (IQR)	0.77 (0.63, 0.98)	0.94 (0.87, 1.5)	0.03^b^	51.5*
Serum albumin (g/dL), median (IQR)	3.1 (2.8, 3.5)	2.6 (2.4, 3)	0.02^b^	168.5*
Serum LDH (U/L), median (IQR)	375 (264, 508)	445 (316, 550)	0.33^b^	83*
Serum CRP (mg/dL), median (IQR)	6.5 (2.36, 8.81)	20.0 (9.5, 27.5)	<0.01^b^	27*
Serum IgG (mg/dL), median (IQR)	1058 (817, 1202)	1059 (939, 1223)	0.91^b^	60.5*
Serum IgA (mg/dL), median (IQR)	250 (184, 315)	184 (153, 189)	0.048^b^	71.5*
Serum KL-6 (U/mL), median (IQR)	720 (312, 1011)	650 (444, 1139)	0.92^b^	99*
Serum SP-D (ng/mL), median (IQR)	142 (97.3, 204.6)	162.7 (79.2, 410.3)	0.46^b^	83*
Plasma (1,3)-β-D-glucan (pg/mL), median (IQR)	48.6 (19.7, 128)	98.7 (16.2, 282.5)	0.69^b^	98*
PaO2/FiO2 ratio (mmHg), median (IQR)	241.0 (165.7, 327.1)	146.5 (52.4, 224.8)	0.02^b^	148.5*
Sputum PCP (PCR), n (%)	18 (58.1)	7 (100)	0.07^a^	-
Treatment of PCP				
ST/ST + pentamidine, n (%)	31 (100)	6 (85.7)	0.18^a^	-
Pulsed-steroid therapy, n (%)	18 (58.1)	7 (100)	0.07^a^	-
Intratracheal intubation, n (%)	10 (32.3)	5 (71.4)	0.09^a^	-
Managing in ICU, n (%)	3 (9.7)	2 (28.6)	0.22^a^	-
Hospitalization (day), median (IQR)	28 (21, 44)	14 (5, 61)	0.13^b^	148.5*

Characteristics of PCP-affected non-survivors

Compared with the survivor group, the non-survivor group had a higher proportion of males, higher levels of creatinine and CRP, as well as lower levels of albumin, IgA, and partial pressure of oxygen/fraction of inspired oxygen (PaO2/FiO2) ratio. No significant difference was observed in the rate of intratracheal intubation, ICU management, and hospitalization period.

## Discussion

Our study investigated the risks for mortality in 38 patients with autoimmune diseases who were diagnosed with PCP. The mortality rate in this study was 18%. Our findings reveal several significant risk factors, including male sex, reduced levels of IgA, albumin, and PaO2/FiO2 ratio, as well as elevated serum creatinine and CRP levels.

Compared with the report by Aoki et al., in which 11 of 25 (44%) patients died [5], the mortality rate in our study was lower, with seven deaths in 38 patients (18%). The low mortality rate was considered to be attributed to three possible reasons. First, the prevalence of lung disease varied. In the report of Aoki et al., lung disease was a comorbidity in 76% of the cases, whereas it was present in 55.2% of patients in our study. In patients with autoimmune diseases, the comorbidity of lung diseases leads to a poor prognosis. For example, Raimundo et al. discovered that over one-third of patients diagnosed with RA-associated interstitial lung disease (RA-ILD) did not survive beyond five years after initial diagnosis. Furthermore, their research revealed that the median life expectancy of patients with RA-ILD in the United States was 7.8 years following the diagnosis of this condition [12]. Second, there were differences in the age of patients among studies. In a previous study, the average age was 58.1 years old, whereas in our study, the patients were older, with an average age of 71.9 years. Dysregulated excessive immunity is considered the pathology of non-HIV cases [13], which may have led to the poorer prognosis observed in younger individuals. Third, the underlying diseases included in this study differed. The prognosis of PCP differs depending on the underlying disease. For example, the mortality rate is 30.8% for RA and 33-57.7% for inflammatory myopathies [3], whereas RA made up 74% in our study. The different compositions of underlying diseases led to the difference in prognosis.

In this study, we identified low IgA levels as a poor prognostic factor. Secretory IgA, the most prevalent immunoglobulin in the human body, plays a crucial role in mucosal immunity, fostering tolerance and safeguarding against infections [14]. A low IgA level increases the risk of infection owing to IgA deficiency [15], in which respiratory infections were particularly prevalent among infections [16]. Additionally, IgA deficiency was associated with autoimmune diseases, including SLE, type 1 diabetes, and RA [17]. Therefore, recognizing the low IgA level, which is more likely to occur in autoimmune diseases, as a risk factor for PCP development is important.

In this study, the median dose of PSL for patients who developed PCP was 5 mg. Previous reports have indicated that the risk increases with a PSL dose of 20 mg or more [18]. However, in our report, PCP developed at a lower dose of PSL, which could be due to the following two reasons. First, the synergistic effect of other risks might be considered. Age, use of immunosuppressive agents, coexistence of autoimmune diseases, and initial PSL dose could have synergistically contributed to the risk. Second, the long-term administration might have an impact. A PSL dose of 20 mg or more for over four weeks has been known to increase the risk of PCP [18]. However, even a low dose of PSL might increase the risk if administered over a long period. Based on the above, prophylactic administration of TMP-SMX may be necessary even for patients with autoimmune diseases receiving low doses of steroids.

In our study, an elevated serum creatinine level was identified as a risk factor for PCP. Patients with chronic kidney disease (CKD) have been reported to have a significantly higher risk of infections compared to the general population [19], with the risk of infection increasing with the severity of kidney impairment [20]. CKD impairs the normal responses of both the innate and adaptive immune systems, increasing the risk of infections and reducing vaccine efficacy, through mechanisms including reduced bactericidal activity of neutrophils, decreased reactivity of monocytes and their differentiation into dendritic cells, reduced T-cell production from the thymus, impaired activation of T-cell responses, increased apoptosis of activated T and B cells, as well as a decrease in B cells [21]. PCP infection is associated with cellular immune deficiency, and the main causes are believed to be the reduction and dysfunction of T cells due to kidney impairment. In addition to the factors discussed above, such as low-dose PSL, age, and low IgA level, a comprehensive evaluation of the overall susceptibility to infections is necessary.

There is a possibility that the administration of salazosulfapyridine had an effect on the onset of PCP and deaths due to PCP. Nunokawa et al. reported that the onset of PCP was reduced by salazosulfapyridine [22]. In our study, although there were no users of salazosulfapyridine at the time of onset of PCP, the use immediately before that is unknown, and the possibility of an effect cannot be denied.

The strength of our study is that we clearly identified the risks that require TMP-SMX prophylaxis. Since our study was conducted in a situation where TMP-SMX was not administered, we believe that we have clarified the patient background that leads to the risk of death from PCP.

This study has some limitations. First, the sample size was small. A multivariate analysis could not be conducted because of the small number of deaths, which may lead to different results if the analysis is performed after adjustment for each factor. Second, it was a single-center study. The practice of diagnosing and treating PCP may influence mortality, and adapting these practices to other facilities may be challenging. Third, a recent change was observed in the proportion of prophylactic administration. Currently, patients at risk are treated with prophylactic administration of TMP-SMX. Therefore, the results of this study may not directly contribute to clinical practice.

## Conclusions

This study examined the risk of mortality for PCP in patients with autoimmune diseases. The male gender, advanced age, low IgA and albumin levels, low PaO2/FiO2 ratio, as well as high creatinine and CRP levels, were identified as significant factors for poor prognosis. These factors can help identify patients at high risk for PCP and guide the consideration of prophylactic TMP-SMX administration. They may also provide insights into when to discontinue prophylactic treatment. However, in autoimmune diseases, the incidence of these risks varies with diseases. Therefore, individual risk assessment is necessary. The small sample size of this study prevents the use of multivariate analysis to weigh these risk factors. Future studies should investigate the administration of prophylactic TMP-SMX. Additionally, the risk of mortality for PCP under the administration of new biological agents, such as anifrolumab, belimumab, and rituximab, or immunosuppressants, such as mycophenolate mofetil and voclosporin, should be evaluated. These findings can contribute to improving PCP prevention and treatment strategies in patients with autoimmune diseases, ultimately leading to better patient outcomes.
